# EphA7 isoforms differentially regulate cortical dendrite development

**DOI:** 10.1371/journal.pone.0231561

**Published:** 2020-12-04

**Authors:** Carrie E. Leonard, Maryna Baydyuk, Marissa A. Stepler, Denver A. Burton, Maria J. Donoghue

**Affiliations:** 1 Department of Biology, Georgetown University, Washington, DC, United States of America; 2 Interdisciplinary Program in Neuroscience, Georgetown University, Washington, DC, United States of America; University of Louisville, UNITED STATES

## Abstract

The shape of a neuron facilitates its functionality within neural circuits. Dendrites integrate incoming signals from axons, receiving excitatory input onto small protrusions called dendritic spines. Therefore, understanding dendritic growth and development is fundamental for discerning neural function. We previously demonstrated that EphA7 receptor signaling during cortical development impacts dendrites in two ways: EphA7 restricts dendritic growth early and promotes dendritic spine formation later. Here, the molecular basis for this shift in EphA7 function is defined. Expression analyses reveal that EphA7 full-length (EphA7-FL) and truncated (EphA7-T1; lacking kinase domain) isoforms are dynamically expressed in the developing cortex. Peak expression of EphA7-FL overlaps with dendritic elaboration around birth, while highest expression of EphA7-T1 coincides with dendritic spine formation in early postnatal life. Overexpression studies in cultured neurons demonstrate that EphA7-FL inhibits both dendritic growth and spine formation, while EphA7-T1 increases spine density. Furthermore, signaling downstream of EphA7 shifts during development, such that *in vivo* inhibition of mTOR by rapamycin in EphA7-mutant neurons ameliorates dendritic branching, but not dendritic spine phenotypes. Finally, direct interaction between EphA7-FL and EphA7-T1 is demonstrated in cultured cells, which results in reduction of EphA7-FL phosphorylation. In cortex, both isoforms are colocalized to synaptic fractions and both transcripts are expressed together within individual neurons, supporting a model where EphA7-T1 modulates EphA7-FL repulsive signaling during development. Thus, the divergent functions of EphA7 during cortical dendrite development are explained by the presence of two variants of the receptor.

## Introduction

In the cerebral cortex, diverse neuronal populations are specifically arranged and this organization underlies remarkable functional complexity [1–4]. Indeed, distinct molecular and morphological characteristics arise during development to determine the function of cortical projection neurons [5,6]. At embryonic timepoints, newly differentiated, bipolar-shaped neurons begin to migrate toward the cortical plate, with a leading process that draws the cell toward the pial surface and a trailing process that extends toward the ventricle [7–9]. This polarity sets the stage for the eventual maturation of the neuron, which is guided by intrinsic and extrinsic programs [reviewed in 6,10]. The trailing process becomes the axon and the leading process develops into the apical dendrite, which will retract or branch extensively, depending on the cell type. Finally, additional basal dendrites emerge from the soma and extend branches, generally in a contact-dependent manner [11–16]. As synaptic connections form and mature during postnatal development, small actin-rich dendritic spines protrude from dendrites, serving as the postsynaptic site for excitatory input [17,18]. Ultimately, dendritic morphology is cell type-specific, serving to integrate signals from specific afferents, and abnormal dendritic morphology has been linked to neurodevelopmental disorders [19–23]. Despite recognition that dendritic form is critical to neuronal function, mechanisms guiding dendrite development remain obscure.

Previously, our research group demonstrated that the intercellular signaling molecule EphA7 plays a variety of roles in cortical neuronal development [24,25]. Membrane-bound Eph receptors engage ephrin ligands on an adjacent cell, activating forward (receptor-mediated), reverse (ligand-mediated), or bi-directional signaling to affect processes in one or both cells [26–28]. Interaction between EphA7 and its high-affinity ligand, ephrin-A5, typically initiates a strong repellent effect between cells [24,29–32]. Accordingly, analyses of cortical neurite extension on patterned substrate revealed that EphA7 mediates dendritic, not axonal, repulsion in response to ephrin-A5. As in other contexts, EphA7-mediated dendrite repulsion requires downstream inhibition of mammalian target of rapamycin (mTOR), since dendrites lacking the mTOR repressor Tsc1 no longer avoid ephrin-A5 [25,33]. Surprisingly, this previous work also demonstrated that EphA7 promotes dendritic spine formation, indicating EphA7 is involved in adhesive intercellular signaling during synaptogenesis [25]. The mechanisms underlying the paradoxical repulsive and adhesive EphA7 functions were unclear.

**Fig 1:**
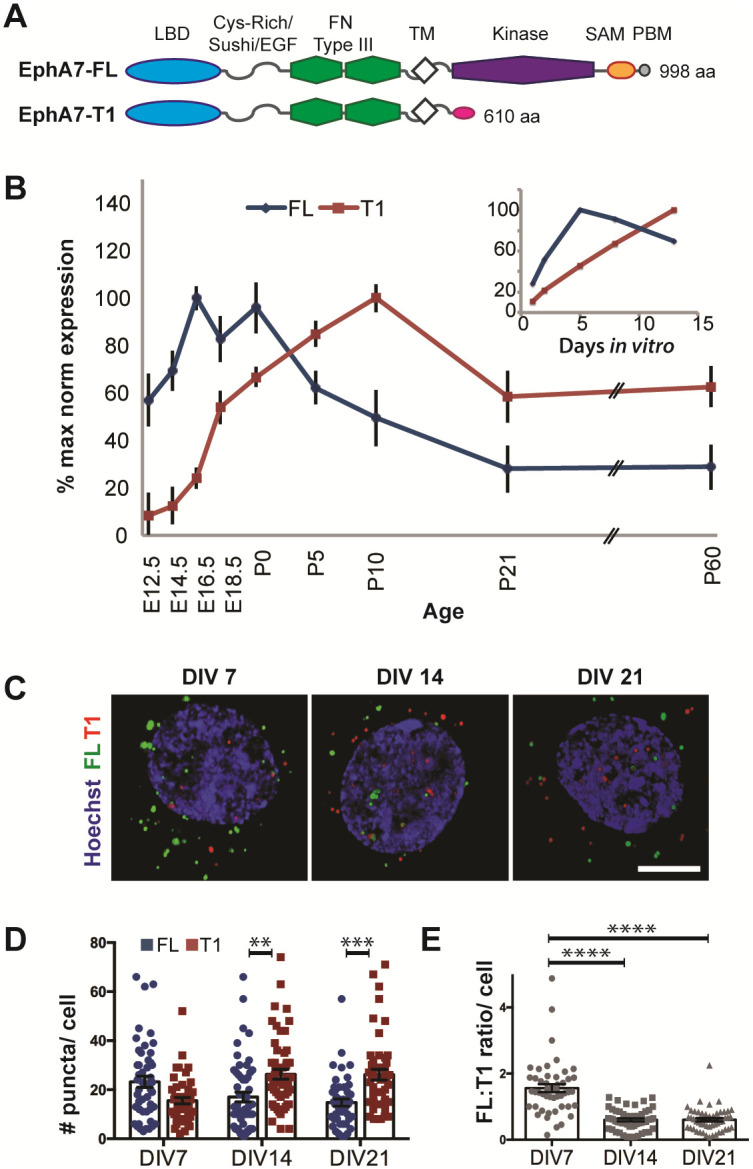
EphA7-FL and EphA7-T1 isoforms have distinct and dynamic expression patterns during cortical development. A. Schematics of EphA7-FL and EphA7-T1 proteins. EphA7-FL (998 aa) and EphA7-T1 (610 aa) have identical N-terminal regions, including ephrin ligand binding domain (LBD), cysteine-rich domain with Sushi and extracellular growth factor-like motifs (Cys-Rich/Sushi/EGF), fibronectin type-III repeats (FN Type III), and transmembrane regions (TM). However, EphA7-FL contains a kinase domain, sterile alpha motif (SAM), and PDZ-binding domain (PBD), while EphA7-T1 has a unique 11 aa C-terminal end (pink). B. Expression profile of EphA7-FL (blue) and EphA7-T1 (red) mRNA transcripts during cortical development (mean ± SEM from 3 animals per timepoint), and in cultured primary cortical neurons (inset, n = 3 cultures each timepoint), obtained with sqPCR and represented as percent maximum expression for each isoform, relative to U6 internal control. C. Fluorescent ISH corresponding to EphA7-FL (green) and EphA7-T1 (red) mRNA at single puncta resolution with the Hoechst-stained (blue) nuclei of primary cortical neurons at 7, 14, or 21 days *in vitro* (Scale bar, 5μm). D. Quantification of mRNA puncta revealed that the average number of EphA7-FL puncta per cell (blue bars) decreased from DIV7 to DIV21, while the average number of EphA7-T1 puncta (red bars) increased over time in culture. On average, there was a trend toward more EphA7-FL than EphA7-T1 puncta at DIV7 (p = 0.06), and there were significantly more EphA7-T1 than EphA7-FL puncta per cell at DIV14 and 21 (n = 60 neurons each timepoint, from three independent experiments, p-values obtained with two-way ANOVA and Tukey correction for multiple comparisons). E. The ratio of EphA7-FL to EphA7-T1 mRNA puncta per cell decreased from DIV7 to 14, with this ratio maintained at DIV 21. (** p<0.01, *** p<0.001, **** p<0.0001, n = 60 neurons per timepoint as in D, p-values from one-way ANOVA with Tukey correction for multiple comparisons).

The current study demonstrates that opposing functions of EphA7 in dendritic repulsion and spine formation are mediated by two distinct isoforms produced by alternative splicing: a full-length EphA7 (EphA7-FL) that includes a kinase domain, and a truncated EphA7 (EphA7-T1) without enzyme activity (Fig 1) [33,34]. Structurally, EphA7-FL and EphA7-T1 have identical extracellular and transmembrane regions, including ligand-binding and other protein interaction domains. Intracellularly, EphA7-FL contains a sterile alpha-motif, PDZ-binding domain, and a kinase domain, which, upon ligand-induced dimerization, is auto-phosphorylated, and initiates downstream signaling. In contrast, EphA7-T1 lacks intracellular signaling domains and, instead, has a unique 11 amino acid C-terminus (Fig 1A) [34]. While previous studies demonstrated that EphA7-FL and EphA7-T1 are both expressed in developing brain, functional consequences of this expression were unknown [34,35–37]. A single study showed that ephrin-A5-expressing non-neuronal cells mixed freely with EphA7-T1-expressing cells, but not with EphA7-FL-expressing cells, and that the presence of EphA7-T1 decreased ligand-induced EphA7-FL phosphorylation [29]. The authors speculated that EphA7-T1 may contribute to adhesive cellular interactions during neural tube closure, acting as an endogenous dominant negative against EphA7-FL [29]. However, this compelling hypothesis remains untested in other systems and a direct interaction between EphA7-FL and EphA7-T1 has yet to be demonstrated.

Here, unique expression patterns of EphA7 isoforms are characterized; expression of EphA7-FL or EphA7-T1 peaks early during dendritic elaboration or later during spine formation, respectively. In addition, increasing relative amounts of EphA7-FL or EphA7-T1 using overexpression produces discrete and opposing effects on dendrite maturation *in vitro*. Moreover, it is revealed that EphA7 downstream signaling may shift between the processes of dendritic repulsion and dendritic spine growth, as inhibition of mTOR via rapamycin ameliorates dendritic branching, but not dendritic spine phenotypes in EphA7-mutant neurons. Finally, direct interaction between EphA7-FL and EphA7-T1 is demonstrated, which causes decreased phosphorylation of EphA7-FL, consistent with EphA7-T1 acting as a dominant negative to attenuate EphA7 repulsive signaling during dendritic spine formation. These results provide mechanistic details of EphA7 signaling during dendritic formation, and highlight the effects of developmentally controlled receptor isoforms in diversifying outcomes.

## Results

### EphA7 isoforms have distinct temporal expression in developing cortex

Cortical neurons in mutant mice lacking all *EphA7* isoforms (*EphA7*^*-/-*^) have longer, more complex dendrites and fewer excitatory synapses compared to cortical neurons in wild type (WT) mice [25]. Previous studies have detected both EphA7-FL and EphA7-T1 (consisting of identical extracellular, but unique intracellular sequences) during normal cortical development, however detailed expression profiles have yet to be described [24,34–36,38]. To understand the developmental expression of EphA7-FL and EphA7-T1 in cortex, semi-quantitative RT-PCR (sqPCR) was performed using isoform-specific primers and values were averaged across 3 animals or 3 culture preparations per time point. The EphA7-FL transcript was highly expressed during cortical development before birth, with expression peaking between embryonic day (E) 16.5 and P0 and decreasing in postnatal cortex (Fig 1B, blue). In contrast, EphA7-T1 transcript levels in the cortex were low throughout the embryonic period, peaked during the early postnatal period with highest levels at P10, then decreased, but remained relatively stable into adulthood (Fig 1B, red). A similar pattern of maturation-dependent isoform expression was observed in samples derived from cultures of primary cortical neurons (Fig 1B, inset).

To determine whether EphA7 isoforms are expressed in the same cells or in discrete populations, RNAscope *in situ* hybridization was used to detect mRNA puncta corresponding to each isoform. Probe specificity was confirmed by comparing fluorescence for each probe in WT versus *EphA7*^*-/-*^ tissue (S1A Fig). EphA7-FL and EphA7-T1 transcripts were examined in individual neurons of primary cortical cultures at 7, 14, or 21 days *in vitro* (DIV, Fig 1C). Immunofluorescent labeling of these cultures with NeuN (neuronal marker) or GFAP (glial marker) demonstrated that EphA7 transcripts were co-localized with NeuN and absent from cells expressing GFAP, indicating that EphA7 is primarily expressed in neurons at these stages (S1B Fig).

Quantification of mRNA puncta corresponding to EphA7-FL and EphA7-T1 transcripts demonstrated that early in cortical neuronal maturation, at DIV7, the average number of puncta per neuron for EphA7-FL trended higher than EphA7-T1 (23 ±2 EphA7-FL puncta compared to 16 ±1 EphA7-T1 puncta per neuron, p = 0.061 calculated via two-way ANOVA with Tukey correction for multiple comparisons, N = 60 neurons from 3 experiments, Fig 1C–1E). The situation was reversed, however, as neurons matured. At both DIV14 and DIV21 the average number of puncta per neuron for EphA7-FL was significantly lower than EphA7-T1 (17±2 EphA7-FL puncta compared to 26 ±2 EphA7-T1 puncta per neuron at DIV14, p = 0.0063; and 15 ±1 EphA7-FL puncta compared to 26 ±2 EphA7-T1 puncta per neuron at DIV21, p = 0.0005, p-values obtained via two-way ANOVA with Tukey correction for multiple comparisons, N = 60 neurons from 3 experiments, Fig 1C–1E). These analyses revealed that neurons positive for one EphA7 isoform also expressed the other, such that every cell expressing EphA7-FL or EphA7-T1 mRNA also expressed the other splice variant. Although there was considerable variation in the amount of EphA7 mRNA expressed per neuron (Fig 1D), the ratio of EphA7-FL to EphA7-T1 mRNA puncta within each individual neuron significantly decreased between DIV7 (1.561 ±0.12) and DIV14 (0.601 ±0.04, p<0.0001) or DIV21 (0.605 ±0.05, p<0.0001 obtained via one-way ANOVA with Tukey’s correction for multiple comparisons, N = 60 neurons from 3 experiments, Fig 1E). The developmental shift in expression was also observed *in vivo* in motor cortex, where expression was high, with EphA7-FL predominantly expressed at P0 and EphA7-T1 preferentially expressed at P10 (S1C–S1E Fig). Taken together, these analyses of EphA7 expression reveal that EphA7-FL and EphA7-T1 are co-expressed within individual neurons, with higher proportions of EphA7-FL expression in immature neurons and more EphA7-T1 as neurons mature.

### Overexpression of EphA7-FL or EphA7-T1 in vitro results in divergent shifts in dendritic complexity and spine density

**Fig 2:**
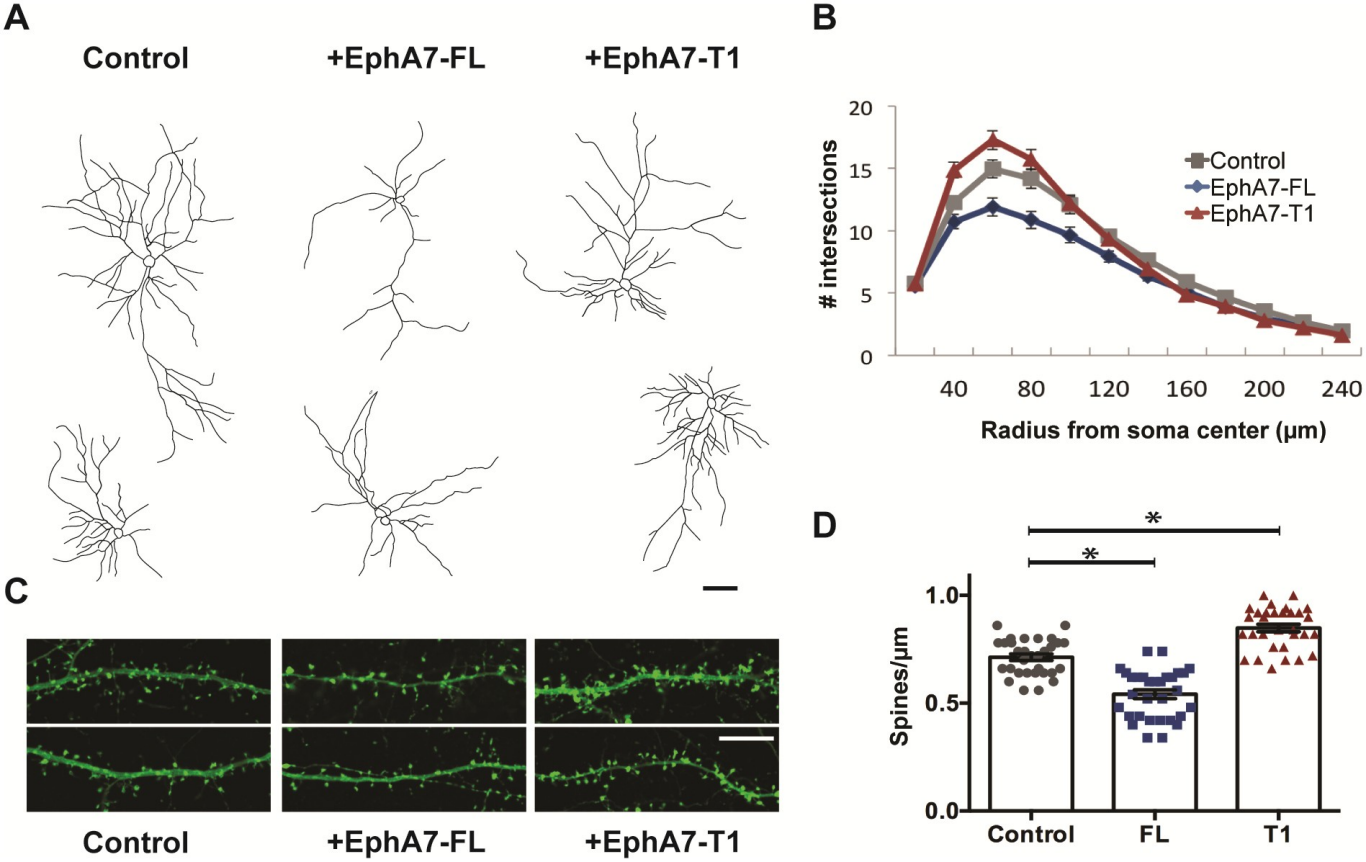
Overexpression of EphA7-FL or EphA7-T1 resulted in distinct shifts in dendritic branching and spine density. Primary cortical neurons transfected at DIV7 with actin-GFP plus control, EphA7-FL-HA, or EphA7-T1-myc plasmids were analyzed at DIV21. A. Representative traces of DIV21 control- (left), EphA7-FL-HA- (middle), or EphA7-T1-myc- (right) transfected cortical neurons (Scale bar, 50μm). B. Data from Sholl analysis of dendrites from primary cortical neurons transfected with actin-GFP plus control (gray), EphA7-FL-HA (blue), or EphA7-T1-myc (red). (Data represented as mean ± SEM from at least 40 neurons per condition across 3 independent experiments). C. Representative images of dendrites with spines from neurons transfected with actin-GFP plus control (left), EphA7-FL-HA (middle), or EphA7-T1-myc (right) (Scale bar, 10μm) D. Compared to control-transfected neurons (gray), EphA7-FL-transfected neurons (blue) had lower dendritic spine density and EphA7-T1-transfected neurons (red) had higher dendritic spine density. (*p<0.0001 using one-way ANOVA with Tukey correction for multiple comparisons, n = 31 neurons per condition from 3 independent experiments).

The discrete temporal expression of EphA7-FL and EphA7-T1 in maturing cortical neurons is consistent with the idea that the isoforms might differentially impact dendritic branching and spine maturation. To examine the functions of each isoform, epitope-tagged EphA7-FL (hemagglutinin for EphA7-FL; EphA7-FL-HA) and EphA7-T1 (myc for EphA7-T1; EphA7-T1-myc) expression constructs were created and their abilities to produce membrane-bound protein capable of binding ephrin-A5 ligand verified (S2 Fig). Then, primary cortical neurons were transfected at DIV7 (empty vector, EphA7-FL-HA or EphA7-T1-myc with actin-GFP to discern morphology). After harvesting neurons at DIV 21 and visualizing GFP, Sholl analysis was performed to quantify the complexity of dendritic arbors. In parallel, dendrites were examined to assess spine density. Compared to control-transfected neurons, dendrites of cells transfected with FL were less complex (Fig 2A and 2B) and had significantly lower dendritic spine density (control = 0.713 ± 0.015 spines/μm; EphA7-FL-transfected = 0.541 ± 0.020 spines/μm, p<0.0001 obtained via one-way ANOVA with Tukey’s correction for multiple comparisons, N≥31 neurons from 3 experiments, Fig 2C and 2D). These results indicate that increasing the ratio of EphA7-FL to EphA7-T1 at DIV7 onward limits both dendritic branching and dendritic spine density in this experimental paradigm. In contrast, compared to control-transfected neurons, EphA7-T1-transfected neurons had dendrites that trended more complex (Fig 2A and 2B) and had significantly higher dendritic spine density (control = 0.713± 0.015 spines/μm; EphA7-T1-transfected = 0.848 ± 0.017 spines/μm, p<0.0001 obtained from one-way ANOVA with Tukey’s correction for multiple comparisons, N≥31 neurons from 3 experiments, Fig 2C and 2D). Thus, increasing EphA7-T1 expression at DIV7 and onward had a modest effect on dendritic complexity but promoted dendritic spine formation. These results demonstrate that EphA7-FL and EphA7-T1 differentially affect dendritic branching and spine density in developing cortical neurons.

### EphA7 downstream signaling varies between elaboration of dendrites and production of spines

**Fig 3:**
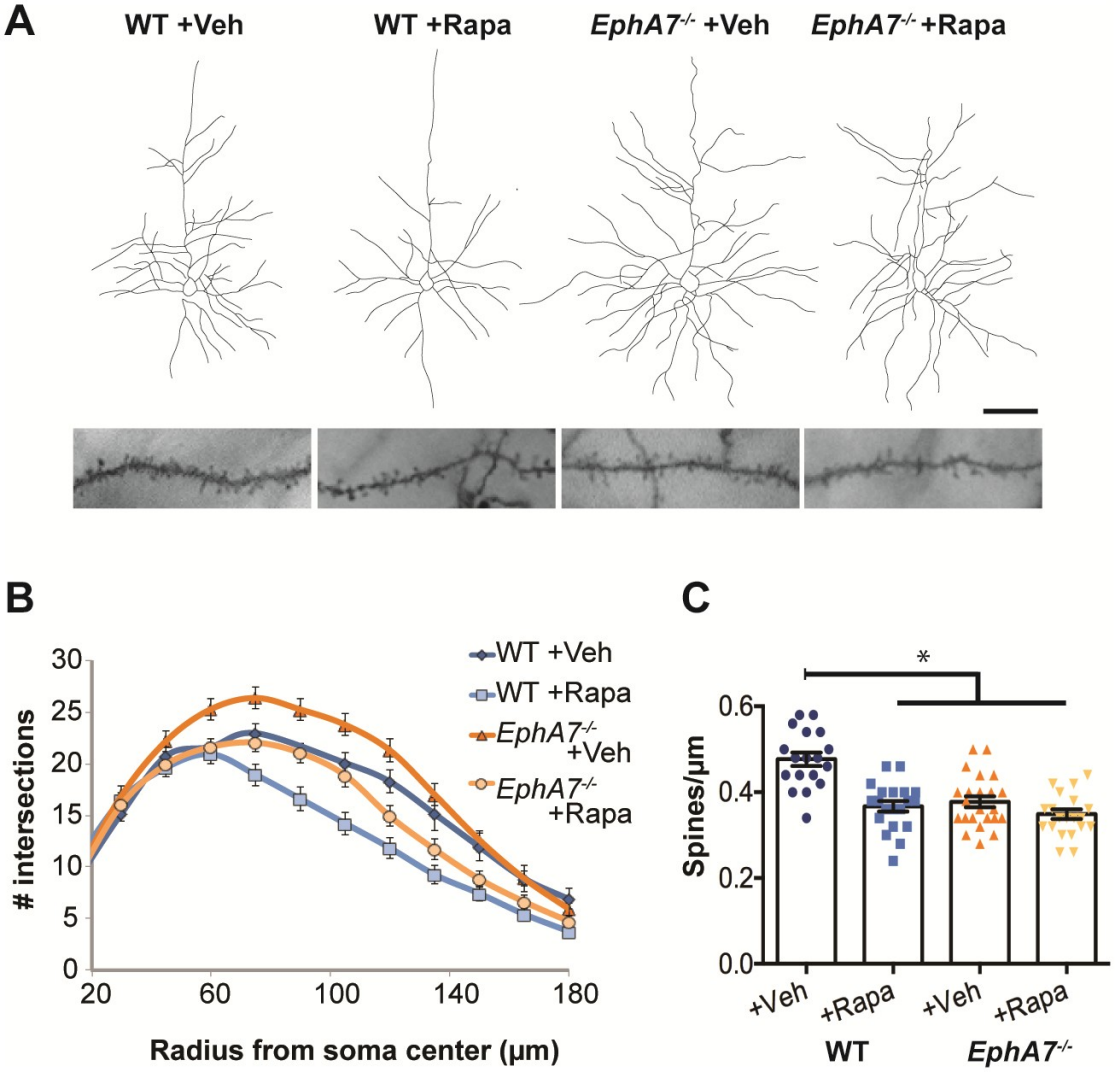
EphA7 downstream signaling varies between elaboration of dendrites and production of spines. A. Representative traces of cells (top) and images of dendrites with spines (bottom) of Golgi-stained deep layer pyramidal neurons from P22 WT (left) or *EphA7*^*-/-*^ (right) animals treated postnatally (from P5-P22) with vehicle (left and second from right) or rapamycin (second from left and right). (Scale bar: 50μm for neuronal traces; 10μm for dendrites). B. Data from Sholl analyses of dendrites from WT/vehicle (darker blue), WT/rapamycin (lighter blue), *EphA7*^*-/-*^/vehicle (darker orange), or *EphA7*^*-/-*^/rapamycin (lighter orange) neurons. (Data are presented as mean ± SEM for at least 30 neurons from at least 6 animals per treatment group.) C. Compared to WT/vehicle neurons (blue), both WT/rapamycin neurons (striped blue) and *EphA7*^*-/-*^/vehicle neurons (orange) had less dense dendritic spines. There was no difference in spine density between vehicle (orange) and rapamycin (striped orange) treated *EphA7*^*-/-*^ neurons. (Data are presented as mean ± SEM for at least 18 neurons from 4–5 animals per treatment group. *p<0.0001 with two-way ANOVA and Tukey correction for multiple comparisons).

Our previous study suggested that dendrite repulsion induced by the EphA7 ligand, ephrin-A5, required downstream inhibition of mTOR via Tsc1, similar to a reported mechanism of EphA-guided axon retraction [25,33]. Since inhibition of mTOR is required for EphA7-mediated repulsive signaling, we explored whether inhibition of mTOR via rapamycin could rescue dendritic phenotypes observed in *EphA7*^*-/-*^ neurons. To this end, WT and *EphA7*^*-/-*^ mice were treated with rapamycin from P5 to P22 and dendritic morphology was visualized using Golgi staining. Consistent with published findings [25], dendrites were more complex in vehicle-treated *EphA7*^*-/-*^ neurons compared to vehicle-treated WT neurons, (Fig 3A and 3B, N≥30 neurons from ≥6 animals per condition). Notably, this phenotype was ameliorated with rapamycin treatment, as dendrites of rapamycin-treated *EphA7*^*-/-*^ neurons were indistinguishable in complexity to vehicle-treated WT neurons within 110 microns of the soma (Fig 3A and 3B). These data indicate the *EphA7*^*-/-*^ dendritic branching phenotype arises, at least in part, from uninhibited mTOR activity in cortical neurons, evidenced by the phenotypic reversal following rapamycin treatment.

The potential for rapamycin to rescue *EphA7*^*-/-*^ dendritic spine phenotype was also examined. *EphA7*^*-/-*^ neurons treated with rapamycin displayed no difference in spine density compared to vehicle-treated *EphA7*^*-/-*^ neurons (*EphA7*^*-/-*^ /veh = 0.38 ± 0.01 spines per micron, *EphA7*^*-/-*^ /rapa = 0.35 ± 0.01 spines per micron, p = 0.366 obtained via two-way ANOVA with Tukey’s correction for multiple comparisons, N≥18 neurons from ≥4 animals per condition, Fig 3A and 3C). However, mTOR inhibition via rapamycin did result in decreased dendritic spine density for WT neurons (WT/veh = 0.48 ± 0.02 spines per micron, WT/rapa = 0.37 ± 0.01 spines per micron, p<0.0001 obtained from two-way ANOVA with Tukey’s correction for multiple comparisons, N≥18 neurons from ≥4 animals per condition, Fig 3A and 3C). Therefore, while mTOR activity appears to be necessary for dendritic spine formation in these cortical neurons, it does not seem to be driving the dendritic spine phenotype observed in *EphA7*^*-/-*^ neurons, like it does for dendritic branching. Together, these results suggest there are discrete EphA7-mediated signaling events during dendritic repulsion (mTOR repression is implicated) versus dendritic spine formation (EphA7-mediated mTOR repression is not apparent).

### EphA7-FL and EphA7-T1 interact to modulate signaling and are co-expressed in cortical synaptosomes

**Fig 4:**
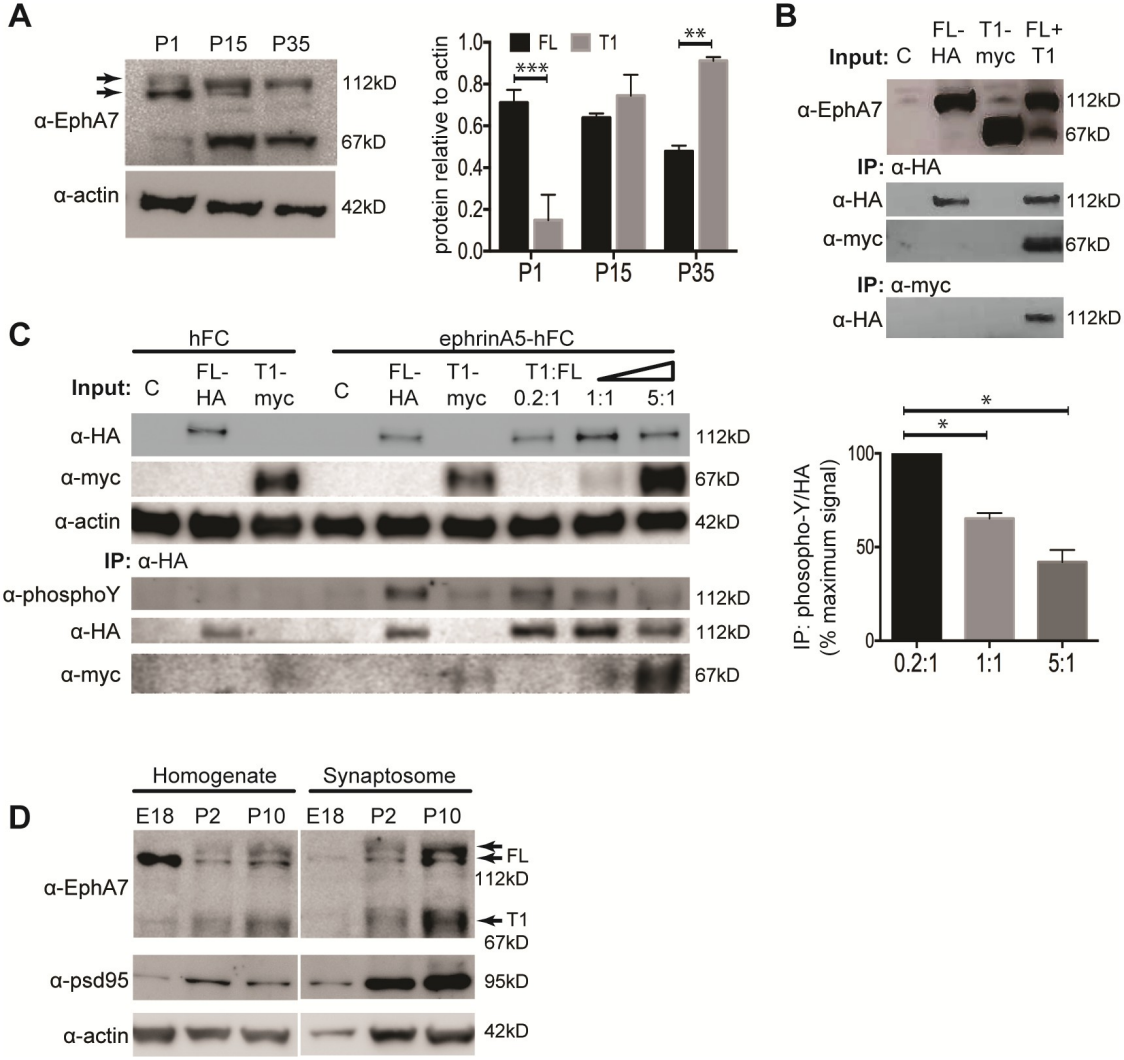
EphA7-FL and EphA7-T1 proteins interact and are colocalized at cortical synapses. A, Representative western blot (left) of EphA7-FL (112 kD predicted) and EphA7-T1 (67 kD predicted) with actin loading control, detected in lysates prepared from P1, P15, and P35 mouse cortex. Quantification and averaging of 2 separate experiments reveals significantly more EphA7-FL compared to EphA7-T1 at P1, and significantly more EphA7-T1 compared to EphA7-FL at P35 (two-way ANOVA with Bonferroni correction for multiple comparisons). B-C, HEK cells were transfected with control, EphA7-FL-HA, EphA7-T1-myc, or both EphA7-FL-HA and EphA7-T1-myc at a 1:1 ratio (B) or at increasing EphA7-T1:FL ratios (C). B. Transfected cells were lysed and immunoprecipitated with HA or myc antibodies; western analysis detecting either total EphA7 or respective epitope tags revealed that EphA7-FL-HA and EphA7-T1-myc protein directly interacts. C. Transfected cells were stimulated for 15 min. with clustered ephrin-A5-hFC, or hFC control. Lysates were subjected to immunoprecipitation with HA antibody to pull down FL protein, followed by detection of phospho-tyrosine, EphA7-T1-myc, and EphA7-FL-HA, which revealed that EphA7-FL tyrosine phosphorylation was decreased with increasing amounts of EphA7-T1 binding. Actin was used as loading control. Quantification (right) of phospho-tyrosine over total HA from 2 separate experiments reveals EphA7-FL-HA tyrosine phosphorylation decreases with increasing amounts of EphA7-T1-myc (one-way ANOVA with Bonferroni correction for multiple comparisons). D. Representative western blot of cortical homogenate and cortical synaptosomes prepared from E18, P2, or P10 rat (all samples were run on the same gel and detected on the same membrane, lanes between homogenate and synaptosomes samples were digitally removed). EphA7 detection in cortical homogenate reveals that the intensity of EphA7-FL bands decreases with developmental time, while the intensity of EphA7-T1 and PSD95 bands increase with time (left). EphA7-FL and EphA7-T1 are detected in P2 synaptosomes, and are enriched in synaptosomes at P10, similar to PSD95 (right). Actin was used as a loading control for homogenates. (*p<0.01, **p<0.005, ***p<0.001).

These data support a scenario where early in cerebral cortical development, EphA7-FL acts in the canonical manner of Eph receptors, involving kinase-dependent forward signaling that inhibits mTOR and initiates cellular retraction, thus restricting dendritic arborization in cortical neurons [25,27,39–42]. There is less clarity on how EphA7-T1, which lacks the intracellular kinase domain, acts in promoting dendritic spines. One study posited EphA7-T1 may act as a dominant negative by heterodimerizing with EphA7-FL, but an interaction was never shown [29]. First, we examined expression of EphA7 protein in mouse cortical lysates from birth to P35 via western blot. Similar to the dynamic transcript expression profile (Fig 1), the ratio of EphA7-FL protein (112 kD) to EphA7-T1 protein (67 kD) decreased over developmental time, such that there was significantly more EphA7-FL protein at P1 (p = 0.0006), but significantly more EphA7-T1 protein at P35 (p = 0.0026 obtained from two-way ANOVA with Bonferroni correction for multiple comparisons, N = 2, Fig 4A). Next, to determine whether the hypothetical interaction between the two isoforms can indeed occur, HEK cells were co-transfected with EphA7-FL-HA and EphA7-T1-myc expression vectors and co-immunoprecipitation of either isoform was performed followed by western blotting for total EphA7 and each respective epitope tag. EphA7-FL and EphA7-T1 did co-immunoprecipitate, confirming that EphA7-FL and EphA7-T1 can directly bind to one another (Fig 4B). To address a proposed dominant negative role, we manipulated the ratio of EphA7 isoforms to mimic the expression profiles in developing cortex (0.2:1, 1:1, and 5:1 ratios of EphA7-T1:FL), then induced tyrosine phosphorylation with ephrin-A5 stimulation. Increasing the EphA7T1:FL ratio to 1:1 or 5:1 resulted in 65% (p = 0.0069) or 42% (p = 0.0015, one-way ANOVA with Bonferroni correction, N = 2) of EphA7-FL tyrosine phosphorylation compared to a ratio of 0.2:1 (Fig 4C) [29]. These results demonstrate that EphA7-FL and EphA7-T1 can assemble when co-expressed in cells, and, under those circumstances, that EphA7-T1 can act to inhibit canonical EphA7 forward signaling by blocking ligand-induced EphA7-FL tyrosine phosphorylation.

Finally, to assess whether EphA7 isoforms are co-expressed within subcellular compartments *in vivo* to facilitate interactions in developing cortical dendrites, levels of EphA7-FL and EphA7-T1 protein were assessed in synaptosomal fractions of E18, P2, and P10 rat cortex. Western blot analysis of cortical homogenates demonstrated that the intensity of EphA7-FL bands decreased over developmental time, while the intensity of the EphA7-T1 band increased during the same period (Fig 4D, left). In cortical synaptosomes, both EphA7-FL or EphA7-T1 were below levels of detection at E18, however both proteins were present at P2 and considerably enriched at P10. Thus, both EphA7-FL and EphA7-T1 are at cortical synapses as they mature and spines become more numerous (Fig 4D, right). Taken together with *in vitro* data that reveal EphA7-FL and EphA7-T1 transcripts are co-expressed within individual neurons (Fig 1), these results provide evidence that EphA7-FL and EphA7-T1 are anatomically positioned in temporally distinct ratios to interact in cortical dendrites and that EphA7-T1 may act as a modulator of EphA7-FL repulsive signaling in developing cortex.

## Discussion

In summary, discrete roles for two EphA7 isoforms, EphA7-FL and EphA7-T1, are described during cortical dendrite development. First, distinct expression patterns exist that correspond with developmental events. EphA7-FL mRNA is most highly expressed in the cortex just before birth, when dendrites are emerging and dendritic spines are sparse. In contrast, EphA7-T1 mRNA expression increases after birth and peaks in the second postnatal week, during rapid synaptogenesis and spine formation. EphA7-T1 transcript levels remain relatively high into adulthood, when dendrites and spines are plastic (Fig 1B). When examining individual cortical neurons *in vitro*, the amount of EphA7 transcripts per cell vary considerably, but EphA7-FL and EphA7-T1 are usually co-expressed within a neuron. Even with such variation, it is interesting that on a cell-by-cell basis, the ratio of EphA7-FL to EphA7-T1 decreases as neurons mature (Fig 1C–1E). When examining expression of EphA7 protein from cortical lysate, a similar dynamic pattern is observed, such that the ratio of EphA7-FL to EphA7-T1 decreases over time, but this shift occurs later in development compared to mRNA changes (Fig 4A). This discrepancy in EphA7 mRNA versus protein expression has been observed in brain previously, and is likely explained by translational or post-translational regulation of EphA7 isoforms, the mechanisms of which have yet to be described. Additionally, EphA7 proteins may have long half-lives with little turnover after the neonatal period [36,37]. Nonetheless, a clear pattern emerges in cortex, even within individual neurons, where the relative amount of EphA7-T1 increases throughout development.

To understand unique functions of EphA7 isoforms, relative amounts of EphA7-FL versus EphA7-T1 were manipulated in cultured cortical neurons. Over-expression of EphA7-FL or EphA7-T1 *in vitro* complemented *in vivo* loss-of-function phenotypes. While deletion of all *EphA7* isoforms results in more complex dendrites and fewer dendritic spines *in vivo*, elevated EphA7-FL expression results in less complex dendrites and fewer dendritic spines, whereas increased EphA7-T1 causes a modest effect on branching but more dendritic spines (Figs 2 and 3) [25]. It is possible that earlier overexpression of EphA7-FL or EphA7-T1 would have augmented their effects, especially on dendritic branching, since primary dendrites are already established and starting to branch by DIV7. However, given EphA7’s roles in other neuronal processes like migration and axon pathfinding, we aimed to target effects to dendrites by choosing this timepoint [31,32,43,44]. Interestingly, most changes in dendritic branching are observed at least 50 microns away from the soma. This is consistent with our previous results that revealed loss of EphA7 affects the number and branching of secondary and tertiary dendrites, but not primary dendrites [25].

*In vivo*, increased dendritic branching caused by loss of EphA7 is ameliorated by the repression of mTOR via rapamycin. Yet, dendritic spine formation is unchanged by rapamycin in *EphA7*^*-/-*^ neurons, indicating the two processes of dendritic branching and spine formation are regulated by EphA7 through different downstream mechanisms (Fig 3). Together these results suggest that activation of EphA7-FL, a receptor tyrosine kinase, normally decreases mTOR activity, thereby inhibiting dendritic growth and branching. Yet dendritic spine formation is likely promoted by EphA7-T1, which lacks a kinase domain and cannot exert the same downstream effects.

Finally, EphA7-FL and EphA7-T1 are shown to directly interact *in vitro*, which can decrease levels of EphA7-FL tyrosine phosphorylation and, therefore, inhibit downstream signaling capabilities (Fig 4B and 4C). Since both EphA7 isoforms are present in cortical synaptic fractions (Fig 4D) and are expressed together within individual neurons (Fig 1), it is likely that EphA7-FL and EphA7-T1 collaborate to mediate signaling in developing cortical neurons. Thus, the paradoxical functions of EphA7 in dendrite maturity are explained through interactions between EphA7-FL and EphA7-T1.

These results expand our understanding of dynamic Eph signaling throughout development. Selective manipulation and detection of native levels of EphA7-FL and EphA7-T1 is a clear next step and would provide valuable insights into isoform function in cortical dendrites and synapses. Unfortunately, several attempts by our laboratory and others’ have not yielded isoform-specific protein reagents and we were not successful in CRISPR-based isoform targeting. The former effort is limited by the highly conserved intracellular sequence of all full-length EphA receptors and by the identical extracellular and transmembrane sequence of EphA7-FL and EphA7-T1, leaving only 11 unique amino acids for specific detection of EphA7-T1. The latter approach is likely impacted by inaccessibility of the EphA7 locus to genomic manipulation, as guide RNAs generated against specific EphA7 isoforms resulted in very low cleavage efficiency. Given these limits, attempts to reduce or detect endogenous EphA7-FL or EphA7-T1 protein are not reliable with current reagents. Although our results align with previous reports of EphA7 protein in cortical neuron dendrites and postsynaptic densities of other neuron types in the brain, technical advances are required to provide further clarity [37,45].

Eph-based signaling is complex, encompassing several ligand-receptor pairs, either uni- and bi-directional signaling, and various downstream signaling targets [26,46,47]. Eph receptors are historically defined by specificity for certain ephrin ligands and there is evidence that some Eph receptors can compensate for the loss of others. However, EphA7, with the highest affinity for and most repulsive response to ephrin-A5, has unique functions in neurite guidance and cell movement which cannot be compensated for by its close relative, EphA4 [30,32,48]. Notably, in cortical neurons, EphA4 specifically regulates axon outgrowth in response to ephrin-A5, whereas EphA7 controls dendrite outgrowth. Therefore, while both EphA7 and EphA4 interact with ephrin-A5, the dendritic responses described in this study are likely unique to EphA7. Moreover, these results focus on changes within receptor-expressing cells. While the extracellular regions of EphA7-FL and EphA7-T1 are identical and both isoforms effectively bind to ephrin-A5, it is possible that ephrin-expressing cells may also be affected by relative changes in EphA7 isoform expression, especially if the presence of EphA7-T1 hinders repulsion between the two cells, which could result in prolonged ligand-based signaling. Future studies are needed to explore these interesting possibilities and overall effects on cortical neuron development.

**Fig 5:**
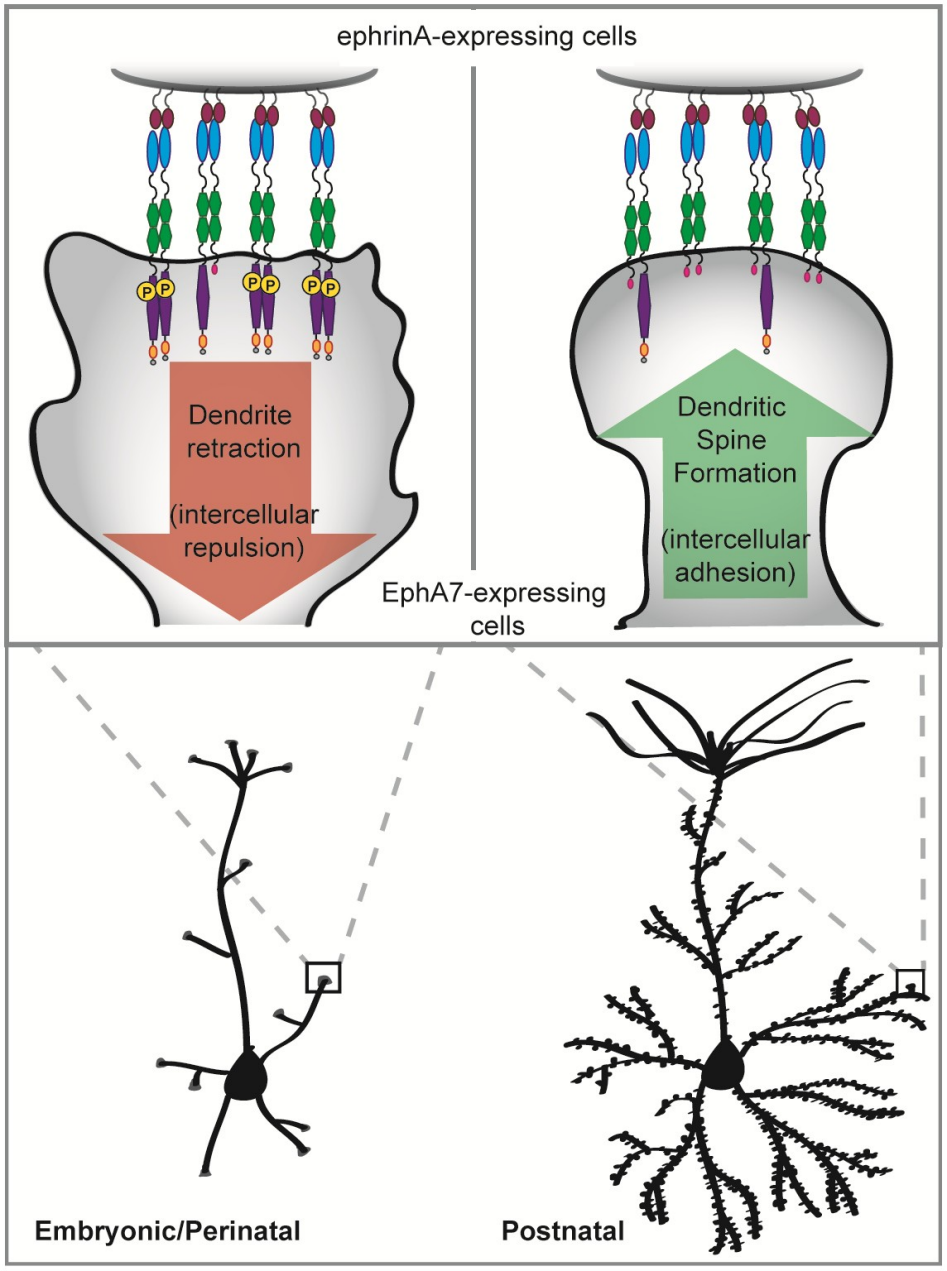
Model of EphA7 function in cortical dendrite development. Our results support a model where full-length (FL) and truncated (T1) EphA7 isoforms have distinct roles in cortical dendrite elaboration and spine formation. As neurons reach their final positions within the cortical plate, they are morphologically simple. Before and around the time of birth, dendrites extend and branch to meet incoming axons (bottom left). During this time, EphA7-FL is highly expressed. Interaction with ephrin-A-expressing cells results in EphA7-FL phosphorylation and initiates forward signaling, which consequently inhibits mTOR activity and dendritic growth (top left). In postnatal cortex, morphologically complex neurons are forming connections (bottom right). EphA7-T1 is highly expressed at this time. Therefore, interaction with ephrin-A-expressing cells no longer initiates repulsive forward signaling, but facilitates intercellular adhesion and synaptogenesis, thus promoting the formation of dendritic spines (top right).

Nonetheless, these results, when taken together, support a model in which EphA7-FL, abundant early in corticogenesis, dimerizes and becomes phosphorylated upon ligand binding, thus activating canonical forward signaling which restricts dendritic outgrowth via repression of mTOR (Fig 5, left). Then, postnatally, EphA7-T1 levels rise, resulting in co-expression with EphA7-FL and likely heterodimers of the two isoforms at cortical synapses. EphA7-FL phosphorylation is inhibited in this context, thus EphA7 repulsive signaling is reduced, which permits the formation of dendritic spines and the synapses they form with other cells (Fig 5, right).

EphA7 joins other examples of endogenous dominant negative regulation in the brain. Notably, a truncated TrkB neurotrophin receptor (TrkB-T1) inhibits full-length TrkB (TrkB-FL) activation by heterodimerization [49,50]. TrkB-T1 also has functions that are independent of TrkB-FL, including BDNF trafficking, neurite extension, and intracellular kinase regulation [reviewed in 51]. Our results do not discount the possibility that EphA7-T1 also has functions independent of EphA7-FL, perhaps even acting in cell adhesion. Since EphA7-T1 is expressed into adulthood, it’s conceivable that it may also be involved in dendritic spine maintenance or activity-dependent signaling. These are interesting possibilities to explore further.

This work highlights the importance of interpreting results with an eye toward the possibility of various receptor and ligand isoforms, including some with divergent outcomes. More broadly, several Eph receptors and ephrin ligands produce alternative mRNA transcripts and proteins of varying sizes: molecular heterogeneity that is largely neglected when assessing Eph/ephrin function [52,53]. Specifically, altered *EPHA7* expression in humans has been implicated in several cancers and neurological deficits, with no distinction as to which isoforms are involved [47,54–58]. The consequences of such molecular diversity warrant attention, especially as Eph receptors are increasingly discussed as potential therapeutic targets for clinical conditions.

## Materials and methods

### Animal husbandry

All animal use and care was in accordance with institutional, Georgetown’s GUACUC, and federal guidelines and the studies described here were approved by GUACUC under protocols #2016–1175 (mice) and #2016–1237 (rats). Control mice, either CD-1 or C57Bl/6, and rats, Sprague Dawley, were purchased from Charles River. Mice mutant for EphA7 [59] were provided by U. Drescher (King’s College, London, UK), were backcrossed onto the C57Bl/6 strain (5–15 generations), and bred as homozygotes as previously described [24,60]. Body sizes of wild type WT and mutant animals treated with rapamycin were similar on the days of analyses. The day of the vaginal plug was considered embryonic day 0.5 (E0.5) and the day of birth, postnatal day 0 (P0). Timed pregnant females were euthanized via CO_2_ asphyxiation followed by cervical dislocation, brains of pups were dissected and either dissociated for cell culture or lysed for protein or mRNA analysis. Postnatal animals were euthanized via hypothermia (under P10) or CO_2_ asphyxiation followed by cervical dislocation (P10 and older) and brains were dissected for protein or mRNA analysis, or fixed, frozen, and sectioned, or were subjected to Golgi staining.

### Plasmids

Plasmids used in these experiments: actin-GFP, described previously [61]; pIRES2-eGFP (referred to as “empty vector” or “control”, Clontech); EphA7-FL-HA (“EphA7-FL-HA”); EphA7-T1-myc (“EphA7-T1-myc”). EphA7-FL-HA and EphA7-T1-myc expression plasmids were created by subcloning cDNA corresponding to either isoform into pIRES2-EGFP (Clontech) and inserting epitope tags via polymerase chain reaction with Pfu DNA polymerase (Promega). Primers for HA insertion into pIRES2-EGFP-FL: forward: 5’-ACCAGACTATGCCCCTGACTTCACTGCCTTCTGTTC-3’, reverse: 5’-ACATCATAGGGATAAGTGCTCTGGTCCAGAAGGAAGC-3’. Primers for myc insertion into pIRES2-EGFP-T1: forward: 5’-ATCTGAAGAGGACTTGTAAACCGCAACAATAACTGTTTAAGAG-3’, reverse: 5’-ATCTGCTTTTGCTCTAAAACTGACAGGTGCTCATTTGTTAC-3’.

### Golgi staining and analyses

Golgi Staining was performed according to manufacturer instructions (FD Neurotechnologies). Briefly, brains from P22 mice were dissected, weighed, rinsed, and incubated in solution A/B for two weeks. Following a week-long clearing step in solution C, brains were frozen in a dry ice/isopentane bath, and sectioned on a cryostat at 120–180μm. Sections were mounted on gelatin-coated slides and dried overnight before being developed in D/E solution, dehydrated, and mounted using Permount (Sigma).

#### Analysis of dendritic extent in vivo

The NeuronJ plug-in [62] for ImageJ (NIH) was used to trace spiny pyramidal neurons in deep layers of mouse motor cortex for examination of dendritic arborization. Two or three neurons were traced from each animal, capturing the entire basal dendritic tree and the first 200 μm of the apical dendrite with oblique branches. Sholl analysis quantified dendritic complexity [63]. Sholl analysis: Concentric circles of increasing radius (15–20μm intervals) were centered on the soma and numbers of dendritic intersections were counted along each circle at a given radius. The average and standard error of the mean (SEM) are reported, referring to the total number of neurons measured from a given group. At least 5 neurons were measured from each of ≥6 animals per treatment group.

#### Analysis of dendritic spine density in vivo

Spiny pyramidal neurons in deep layers of mouse motor cortex were examined. For each cell, a 50 μm segment of a secondary basal or apical oblique branch was identified, the number of dendritic spines within each segment was recorded, and density was calculated as number of dendritic spines divided by 50 μm. Average and SEM are reported, referring to the total number of cells analyzed within a given group. At least 4 neurons were analyzed from each of 4–5 animals per treatment group. Comparisons were made using two-way (WT/veh vs WT/rapa vs *EphA7*^*-/-*^ /veh vs *EphA7*^*-/-*^ /rapa) ANOVA, followed by Tukey’s correction for multiple comparisons (GraphPad Prism 6).

### In vivo rapamycin treatment

Before each injection, animals were weighed and treatment doses were prepared (6 mg/kg) by diluting in sterile vehicle (2.5mL 5% polyethylene glycol, 2.5mL 5% Tween-80, and 45mL sterile, deoionized water, filtered with Steriflip (Millipore)) in accordance with previously published studies at similar ages [64–66]. WT or *EphA7*^*-/-*^ pups were treated with rapamycin (LC labs) via intraperitoneal injection 3 days per week (MWF) from P5-6 until P22-23, when they were euthanized. Upon euthanization, animals were weighed, and brains were dissected and subjected to Golgi staining. Consistent with other reports in young mice, rapamycin-treated animals weighed less than vehicle-treated animals, with no significant differences in brain weight [67]. There were no differences in weight due to genotype during the course of the experiment (S3 Fig).

### Primary cell culture

Primary cortical neurons were prepared from E18.5 rat embryos. One day before culture, acid-washed, glass coverslips were coated with 100 μg /mL poly-d-lysine in sterile PBS and incubated overnight at room temperature. The next day, coverslips were washed with sterile water prior to plating. An E18.5 pregnant rat was euthanized with CO_2_ asphyxiation and pups were collected. Dorsal cortex was immediately dissected in 1x HBSS+ (Hank’s Balanced Salt Solution plus 10mM HEPES and 1% penicillin/streptomycin). Chopped tissue was washed in fresh HBSS^+^, then incubated in 0.125% trypsin/HBSS+ for fifteen minutes at 37° Celsius. Tissue was rinsed three times with HBSS and triturated in neuronal growth media (Neurobasal supplemented with 2% B27, 1% Glutamax, and 1% penicillin/streptomycin). Cells were plated at a density of 150,000 cells per well of a 12-well dish and maintained at 37° C, 5% CO_2_ in a humidified environment. Cultures were fed by replacing half of the media volume with fresh growth media twice per week.

### Cell line culture

HEK293 cells were grown in DMEM (Dulbecco’s Modified Eagle Medium) supplemented with 10% fetal bovine serum and 1% penicillin/streptomycin, and maintained at 37° C with 5% CO_2_ in a humidified environment. Cells were passaged at no more than 80% confluency by brief trypsinization at room temperature, followed by serum inactivation and plating in fresh growth media.

### Semi-quantitative RT-PCR

Total RNA was isolated from cerebral cortical tissue of E12.5, E14.5, E16.5, E18.5, P0, P5, P10, P21 and P60 mice or from cultures of mouse primary cortical neurons at days *in vitro*(DIV) 1, 2, 3, 5, 8, 10, and 13 using the Tri-Regent Kit (Sigma-Aldrich). cDNA was synthesized using the First-Strand cDNA Synthesis Kit (Invitrogen). Primers specific for mouse EphA7-FL, EphA7-T1, and U6 (EphA7-FL forward: 5′-CGTCTGAAGATGCTGGTGAAAGCA-3′, EphA7-FL reverse: 5′-ACTGAAGCTCTTGATGTGCTCGGA-3′; EphA7-T1 forward: 5′-TAAGGACCTGGATTCCTTCAGCGA-3′, EphA7-T1 reverse: 5′-CTGCATGCTTCTGGGTGCATTGAA-3′; U6 forward: 5′-CTCGCTTCGGCAGCACA-3′, U6 reverse: 5′-AACGCTTCACGAATTTGCGT-3′) were designed and used in semi-quantitative real-time RT-PCR (sqPCR). sqPCR was performed using the 2× SYBR Green PCR Master Mix (Bioline) with the CFX96 detection system and analyzed with CFX Manager softward (Bio-rad). Transcript levels were normalized to U6 and converted to percent maximum relative expression for EphA7-FL and EphA7-T1 separately. Each individual experiment was performed with experimental triplicates and each timepoint was analyzed from three separate animals. Reported values are the average and SEM of 3 experiments (animals).

### Transfection

#### HEK cell transfection

HEK cells were transfected at 50–60% confluent in complete growth medium, using X-tremeGENE (Roche), according to manufacturer’s instructions. For one well of a 6-well dish, 2 μg total DNA was transfected at 1:1 DNA:reagent ratio, where 0.5 μg /well of each expression plasmid was added plus empty vector to total 2 μg. Transfection cocktail was added directly to the well and left until cell lysis.

#### Neuron transfection

Primary cortical neurons were transfected at DIV7 with Lipofectamine 2000 (ThermoFisher). One day before transfection, conditioned media was prepared by removing half the volume of media per well and mixing with equal volume fresh neuronal growth media, then supplementing the neurons with fresh media. On the day of transfection, transfection cocktails were prepared according to manufacturer instructions, by mixing (per well of 12-well dish) 200uL non-supplemented Neurobasal media, 0.1 μg /kB of desired expression plasmid(s), and 1uL transfection reagent. Transfection cocktails were added to neurons and allowed to rest at 37° C Celsius for 60–90 minutes. Transfection media was completely removed and replaced with conditioned media, and neurons were returned to 37° C.

### Ligand binding assay

To determine whether EphA7 expression constructs bind to ephrin ligand, HEK cells were transfected with empty vector, EphA7-FL-HA, or EphA7-T1-myc. After 24hrs, cells were fixed in 4% paraformaldehyde for 20 minutes, then rocked at room temperature for at least one hour, while incubating with ephrin-A5 conjugated to human FC (R&D) at a concentration of 3mg/ml in 0.5x blocking buffer (0.01% Triton X-100, 1% BSA) in PBS. Cells were fixed again and subjected to immunocytochemistry [39].

### Fluorescent immunocytochemistry

The following primary antibodies were used: rabbit anti-GFP (ThermoFisher, 1:3000 dilution); rabbit anti-NeuN (CST, 1:1000); rabbit anti-GFAP (CST, 1:1000); mouse anti-PSD95 (Sigma, 1:1000); rabbit anti-HA (CST, 1:1000); rabbit anti-myc (CST, 1:1000); goat anti-EphA7 (R&D, 1:500); goat anti-human (ThermoFisher, 1:1000). Hoechst staining (ThermoFisher, 1:15,000) was also performed. Appropriate Alexafluor-conjugated secondary antibodies (ThermoFisher) were used at a dilution of 1:500. For live imaging of transfected HEK cells, goat anti-EphA7 (R&D) was diluted 1:500 in pre-warmed, serum free DMEM and growth media was replaced with anti-EphA7/media cocktail for at least two hours at 37°C before fixation and application of secondary antibody. After their respective treatments, cells were fixed for 20 min with 4% paraformaldehyde/4% sucrose at room temperature. Cells were permeabilized in 0.1% Triton X-100 in PBS, before blocking in 0.1% Tx-100 and 10% BSA in PBS. Cells were incubated with primary antibody diluted in 0.1% Tx-100 and 1% BSA in PBS overnight at 4°C, then with secondary antibody for at least 1 hour at room temperature before brief application of Hoechst stain in PBS, followed by rinsing and mounting with Fluoromount-G (SouthernBiotech).

### In vitro analysis of dendritic extent and dendritic spine density

Neuron morphology was visualized by ICC for GFP. Neurons were selected for analysis if they were spiny, and displayed 4–7 primary dendrites, with a prominent “apical” dendrite. Three independent experiments were performed from separate culture preparations.

#### Analysis of dendritic extent

Sholl analysis was performed as described above. The mean and standard error of the mean (SEM) are reported for at least 40 neurons per condition from three independent experiments, each with duplicate cultures.

#### Analysis of dendritic spine density

Dendritic spine density was calculated as described above. Mean and SEM are reported for at least 31 neurons per condition from three independent experiments, each with duplicate cultures. Comparisons were made using one-way ANOVA, followed by Tukey’s correction for multiple comparisons (GraphPad Prism 6).

### RNAscope multiplex fluorescent in situ hybridization assay

RNAscope probes were designed to detect mouse EphA7 mRNA for either full length (FL, Mm-Epha7-tv1tv3, targeting sequence: 2189–3239 of NM_010141.4) or truncated isoform (T1, Mm-Epha7-tv2, targeting sequence: 2064–3253 of NM_001122889.1). RNAscope Multiplex Fluorescent Assay on cultured cells and fixed frozen tissue sections was performed according to manufacturer’s instructions (Advanced Cell Diagnostics, ACD, Hayward, CA). Dissociated cortical neurons were cultured on glass cover slips and fixed in 4% formaldehyde solution for 30 min (room temperature) at DIV 7, 14, and 21. Fixed cells were dehydrated and stored in 100% ethanol at -20°C for up to 6 months. Prior to hybridization, cells were rehydrated, permeabilized with 1x PBS containing 0.1% Tween-20 for 20 min and treated with protease III (1:10, ACD) for 20 min. For RNAscope fluorescent assay on fixed frozen tissue, the slides containing 12.5 μm -thick coronal sections of mouse brains, collected at P0 and P10 were submerged into boiling 1x target retrieval buffer (ACD) for 5–10 min, followed by protease III treatment (ACD) for 30 min at 40°C. Following ACD protocol for hybridization and signal amplification steps, the cover slips with cultured cortical cells or slides with brain tissue were either mounted with Fluoromount-G (SouthernBiotech) or subjected to a subsequent immunocytochemistry. Images were acquired with the same parameter settings using Zeiss LSM 880 confocal microscope. mRNA puncta were quantified using Imaris software (Bitplane, Oxford Instruments). For cortical culture, 20 neurons per 3 separate experiments were analyzed for each time point in vitro, final N = 60. For in vivo experiments, mRNA puncta were quantified in sections containing motor cortex, 2–3 sections per animal, 3–4 animals per age group. Comparisons between EphA7-FL and EphA7-T1 in all cases were made using two-way ANOVA, followed by Holm-Sidak (in vivo) or Tukey’s (in vitro) corrected post-hoc analysis. Comparison of FL:T1 ratio per cell was made using one-way ANOVA, followed by Tukey’s correction for multiple comparisons.

### Co-immunoprecipitation Assay and ephrin-A5 treatment in HEK cells

For co-immunoprecipitation, protein A/G agarose beads (Santa Cruz) were washed in immunoprecipitation buffer (1% NP-40, 50 mm Tris-HCl, pH 8.0, 150 mm NaCl) and incubated with primary antibody at 1:200 dilution (rabbit anti-HA or anti-myc, CST) for ≥1hr, then rinsed in IP buffer. For ephrin-A5 treatment (Fig 4B), ephrin-A5 conjugated to human FC (ephrin-A5-hFC, R&D) or hFC control (R&D) was pre-clustered with goat anti-human antibody (Abcam) for one hour at 37°C before being added to transfected HEK cells (5 μg /ml) for 15 minutes. Treated or untreated transfected HEK cells were lysed in cold IP buffer with protease inhibitors (Roche), and centrifuged for 10 minutes and 5,000 rpm at 4°C. Supernatant was collected and measured via Bradford Protein Assay (Bio-rad). 200 μg total protein was incubated with prepared agarose beads in IP buffer overnight at 4°C. Samples were washed in cold IP buffer before being eluted in 2X Laemmli sample buffer with DTT, boiled, and separated via SDS-PAGE.

### Cortical lysate and synaptosomal fractionation

Crude synaptosomal fractions were collected from embryonic or postnatal rat cortex using the Syn-PER Extraction Reagent (ThermoSci) according to manufacturer’s directions. Briefly, dissected cortical tissue was weighed and gently homogenized in ice cold Syn-PER reagent plus protease inhibitors (10mL/gram of tissue). Samples were centrifuged at 1200 x *g* for 10 minutes at 4°C and supernatants were collected (homogenate sample). For synaptosomes, samples were centrifuged again at 15,000 x *g* for 20 minutes at 4°C and the pellet was resuspended in Syn-PER reagent (synaptosome sample) or the supernatant was collected (cytosolic sample). Samples were mixed with 2X Laemmli sample buffer with DTT, boiled, and separated via SDS-PAGE. For analysis of protein in mouse whole cortical lysate in Fig 4A, mouse cortices were collected at P1, P15, and P35 and homogenized in cold IP buffer with protease inhibitors and clarified via centrifugation as described above.

### Western blotting

Cell lysates were collected as described and denatured by boiling in Laemmli sample buffer with DTT before SDS-PAGE separation on 12% pre-cast gels (Bio-rad). Western blots were performed using the Bio-Rad Mini TransBlot and TransBlot-Turbo Transfer System, with the Bio-Rad PVDF Transfer Kit. Chemiluminescent imaging was performed on the ImageQuant LAS4000 biomolecular imager (GE Healthcare). The following primary antibodies were used: rabbit anti-EphA7 (Santa Cruz, 1:500), mouse anti-HA (Sigma, 1:1000), rabbit anti-HA (CST, 1:1000), mouse anti-myc (Sigma, 1:1000), rabbit anti-myc (CST, 1:1000), mouse anti-actin (Sigma, 1:2500), rabbit anti-phospho-tyrosine (CST, 1:1000), mouse anti-psd95 (CST, 1:1000), rabbit anti-Akt(pan) (CST, 1:1000). Appropriate HRP-conjugated secondary antibodies (Jackson ImmunoResearch) were used at a dilution of 1:2500. When appropriate, bands were quantified via densitometry with GelQuant software (BiochemLab Solutions), averaged from two experiments, and compared using one- or two-way ANOVA with Bonferroni’s multiple comparisons correction (Graphpad Prism 6).

## Supporting information

S1 Fig EphA7-FL and EphA7-T1 transcripts are dynamically expressed in neurons throughout the developing cortex. A. Specificity of EphA7-FL (left) and EphA7-T1 (right) RNAscope ISH probes was verified, evidenced by lack of fluorescent signal in *EphA7*^*-/-*^ (bottom) motor cortex compared to WT (top) motor cortex from P10 mice (Scale bar, 50μm.) B. ISH for EphA7-FL (green) and EphA7-T1 (white) mRNA with immunolabeling of NeuN (red, top) or GFAP (red, bottom) in DIV7, 14, or 21 primary cortical neurons reveals EphA7-FL and EphA7-T1 mRNA is primarily present in NeuN-positive cells, and absent from GFAP-positive cells (Scale bar, 10μm) C. Low magnification image of ISH for EphA7-FL (green) in P0 or P10 WT motor cortex. Examples of cortical bins (P0, left) or layers (P10, right) based on nuclear density are shown, which were used to quantify EphA7-FL or EphA7-T1 puncta per 100μm ^2^ in D-E (Scale bar, 200μm for both panels). D. Fluorescent ISH for EphA7-FL (green, left) and EphA7-T1 (red, right) mRNA in motor cortex at P0 (top) or P10 (bottom) (Scale bar, 25μm). A-D, all nuclei were visualized by Hoechst staining (blue). E. Quantification of EphA7-FL (blue) or EphA7-T1 (red) puncta in motor cortex of P0 (top) or P10 (bottom) mice. At P0, there were more EphA7-FL than EphA7-T1 puncta in superficial bins (2 and 3), while EphA7-FL and EphA7-T1 counts were not different in the most superficial (1) and deep bins (4, 5). At P10, EphA7-T1 puncta were more abundant in every layer except for superficial layer I. (** p<0.01, *** p<0.001, **** p<0.0001, n = 3–4 animals for each time point, 3 serial sections per animal. Abbreviations: CP, cortical plate; IZ, intermediate zone; VZ, ventricular zone; SVZ, subventricular zone; Ctx, cortex; CC, corpus callosum; Str, striatum).
(TIF)

S2 Fig Validation of EphA7-FL-HA and EphA7-T1-myc expression constructs. A. Schematic of EphA7-FL-HA (top) and EphA7-T1-myc (bottom) protein. Each construct expresses proteins with the respective EphA7-FL or EphA7-T1 domains described in Fig 1, with an additional C-terminal epitope tag: HA (green) for EphA7-FL, and myc (yellow) for EphA7-T1. B-E, HEK cells were transfected with GFP plus control, EphA7-FL-HA, or EphA7-T1-myc expression constructs for 24hrs. B. Western blot on cell lysates to detect EphA7, HA, or myc revealed the expression constructs produced EphA7 isoforms of the correct sizes (FL, predicted 112kD; EphA7-T1 predicted 67kD) that were successfully epitope tagged. C. Transfected cells were fixed for immunocytochemistry against HA (red, left) or myc (red, right), which indicated the majority of GFP-positive cells (green) were also positive for HA or myc when EphA7-FL-HA or EphA7-T1-myc was co-transfected, respectively. D. Live transfected cells were incubated with EphA7 antibody that recognizes the extracellular portion of the protein in order to determine whether EphA7-FL-HA and EphA7-T1-myc were localized to the cell membrane. Cells transfected with EphA7-FL-HA or EphA7-T1-myc had high anti-EphA7 signal (red), indicating the protein produced from these constructs is trafficked to the membrane. E. Transfected cells were fixed, incubated with ephrin-A5 conjugated to human FC, then fixed once more to determine if EphA7-FL-HA or EphA7-T1-myc can readily bind to ligand. Anti-human immunocytochemistry (red) revealed that indeed, both EphA7-FL-HA and EphA7-T1-myc robustly bind to ephrin-A5. For C-E, nuclei were detected with Hoechst staining (blue) (Scale bars for all panels represent 30μm).
(TIF)

S3 Fig Effect of rapamycin treatment on WT and EphA7^-/-^ body weight, brain weight, and dendritic spines. A. Growth curves of WT/vehicle (dark blue, n = 7), WT/rapamycin (light blue, n = 8), *EphA7*^*-/-*^/vehicle (dark orange, n = 9), and *EphA7*^*-/-*^/rapamycin (light orange, n = 10) animals. B. WT animals treated with rapamycin (6.29g ± 0.25g, blue stripe) weighed less than WT animals treated with vehicle (10.99g ± 0.53g, blue, p<0.0001) and *EphA7*^*-/-*^ animals treated with rapamycin (7.49g ± 0.32g, orange stripe) weighted less than *EphA7*^*-/-*^ animals treated with vehicle (11.98g ± 0.73g, orange, p<0.0001). There were no differences due to genotype. C. There were no differences in brain weight between WT/vehicle (0.37g ± 0.03g), WT/rapamycin (0.31g ± 0.02g), *EphA7*^*-/-*^/vehicle (0.34g ± 0.02g), and *EphA7*^*-/-*^/rapamycin (0.32g ± 0.01g) animals. D. The average brain weight to body weight ratio increased in rapamycin-treated WT (0.050 ± 0.003, blue stripe) and *EphA7*^*-/-*^ (0.044 ± 0.002, orange stripe) animals compared to vehicle-treated WT (0.034 ± 0.003, blue) or *EphA7*^*-/-*^ (0.029 ± 0.002, orange) animals. There were no differences due to genotype. E. There were no differences between dendritic spine densities counted from apical oblique (AO) versus basal dendritic branches, therefore total dendritic spine density is reported in Fig 3. (**p<0.01, ***p<0.001, ****p<0.0001).
(TIF)

S1 Raw Images (PDF)
