# Supplementary figures and images for: EphA7 isoforms differentially regulate cortical dendrite development
Source: PLoS One. 2020 Dec 4;15(12):e0231561. doi: 10.1371/journal.pone.0231561 (PMC7717530; doi:10.1371/journal.pone.0231561)

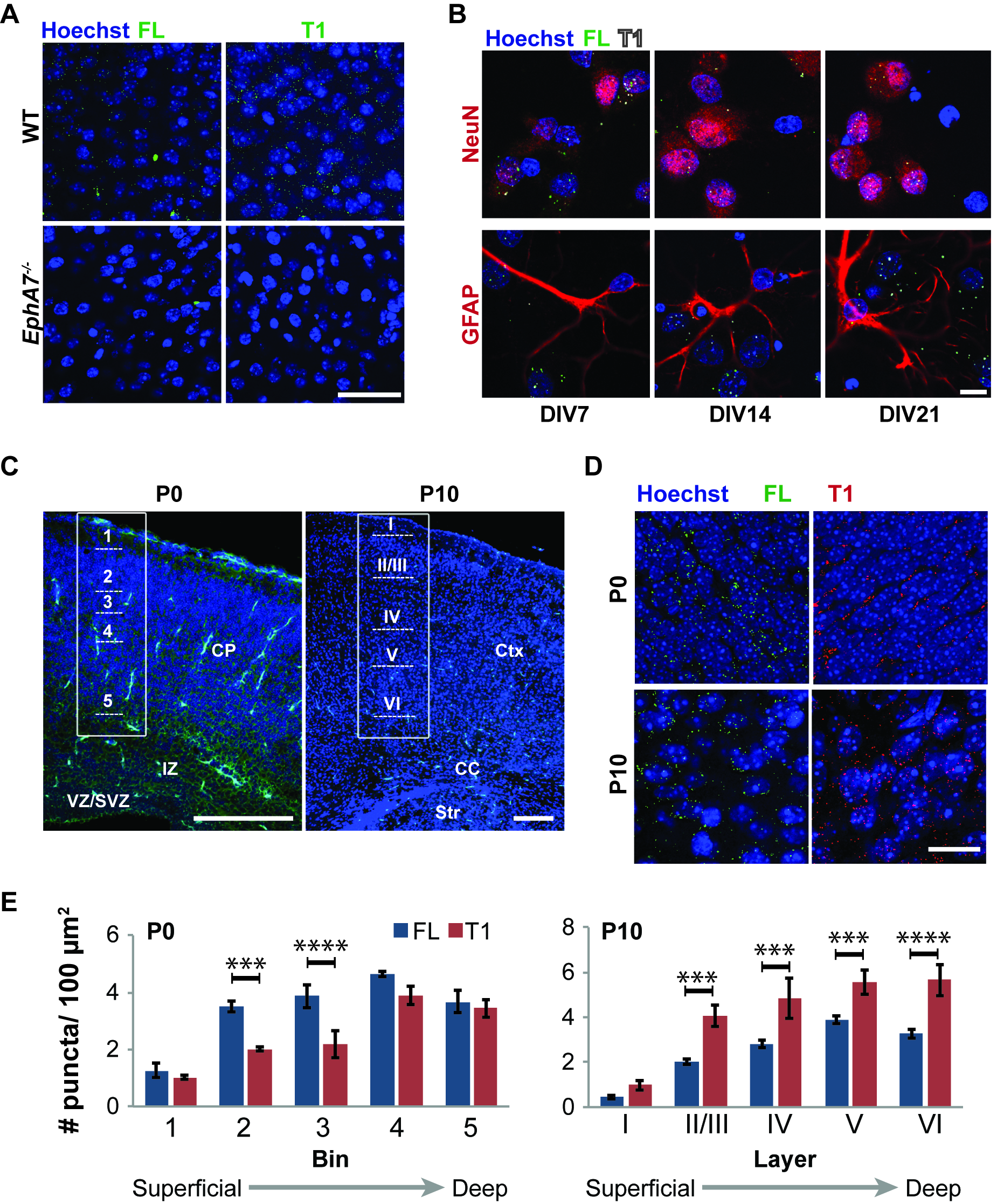

Supplement: S1 Fig — A. Specificity of EphA7-FL (left) and EphA7-T1 (right) RNAscope ISH probes was verified, evidenced by lack of fluorescent signal in EphA7-/- (bottom) motor cortex compared to WT (top) motor cortex from P10 mice (Scale bar, 50μm.) B. ISH for EphA7-FL (green) and EphA7-T1 (white) mRNA with immunolabeling of NeuN (red, top) or GFAP (red, bottom) in DIV7, 14, or 21 primary cortical neurons reveals EphA7-FL and EphA7-T1 mRNA is primarily present in NeuN-positive cells, and absent from GFAP-positive cells (Scale bar, 10μm) C. Low magnification image of ISH for EphA7-FL (green) in P0 or P10 WT motor cortex. Examples of cortical bins (P0, left) or layers (P10, right) based on nuclear density are shown, which were used to quantify EphA7-FL or EphA7-T1 puncta per 100μm 2 in D-E (Scale bar, 200μm for both panels). D. Fluorescent ISH for EphA7-FL (green, left) and EphA7-T1 (red, right) mRNA in motor cortex at P0 (top) or P10 (bottom) (Scale bar, 25μm). A-D, all nuclei were visualized by Hoechst staining (blue). E. Quantification of EphA7-FL (blue) or EphA7-T1 (red) puncta in motor cortex of P0 (top) or P10 (bottom) mice. At P0, there were more EphA7-FL than EphA7-T1 puncta in superficial bins (2 and 3), while EphA7-FL and EphA7-T1 counts were not different in the most superficial (1) and deep bins (4, 5). At P10, EphA7-T1 puncta were more abundant in every layer except for superficial layer I. (** p<0.01, *** p<0.001, **** p<0.0001, n = 3–4 animals for each time point, 3 serial sections per animal. Abbreviations: CP, cortical plate; IZ, intermediate zone; VZ, ventricular zone; SVZ, subventricular zone; Ctx, cortex; CC, corpus callosum; Str, striatum). (TIF) [file pone.0231561.s001.tif]

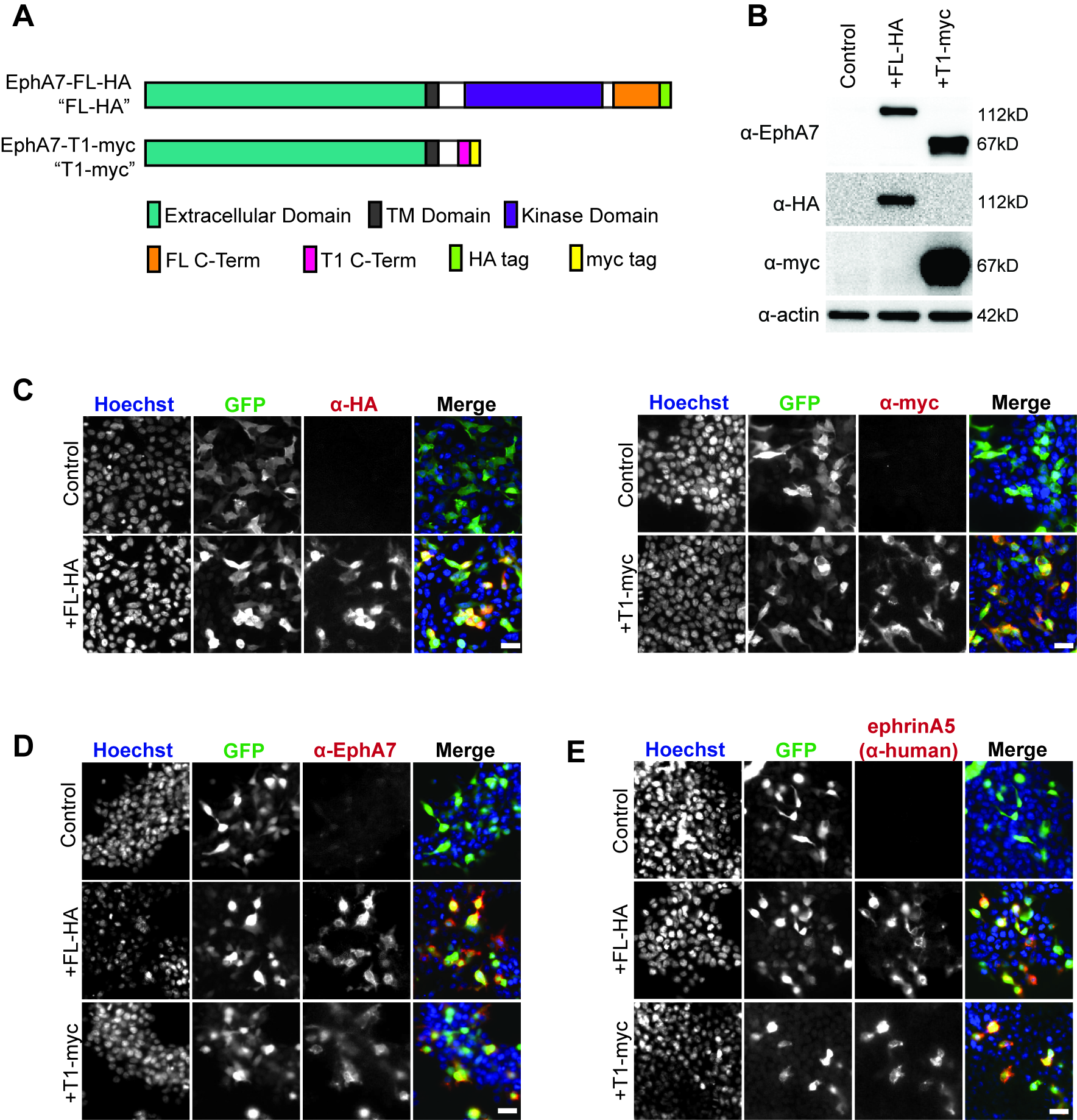

Supplement: S2 Fig — A. Schematic of EphA7-FL-HA (top) and EphA7-T1-myc (bottom) protein. Each construct expresses proteins with the respective EphA7-FL or EphA7-T1 domains described in Fig 1, with an additional C-terminal epitope tag: HA (green) for EphA7-FL, and myc (yellow) for EphA7-T1. B-E, HEK cells were transfected with GFP plus control, EphA7-FL-HA, or EphA7-T1-myc expression constructs for 24hrs. B. Western blot on cell lysates to detect EphA7, HA, or myc revealed the expression constructs produced EphA7 isoforms of the correct sizes (FL, predicted 112kD; EphA7-T1 predicted 67kD) that were successfully epitope tagged. C. Transfected cells were fixed for immunocytochemistry against HA (red, left) or myc (red, right), which indicated the majority of GFP-positive cells (green) were also positive for HA or myc when EphA7-FL-HA or EphA7-T1-myc was co-transfected, respectively. D. Live transfected cells were incubated with EphA7 antibody that recognizes the extracellular portion of the protein in order to determine whether EphA7-FL-HA and EphA7-T1-myc were localized to the cell membrane. Cells transfected with EphA7-FL-HA or EphA7-T1-myc had high anti-EphA7 signal (red), indicating the protein produced from these constructs is trafficked to the membrane. E. Transfected cells were fixed, incubated with ephrin-A5 conjugated to human FC, then fixed once more to determine if EphA7-FL-HA or EphA7-T1-myc can readily bind to ligand. Anti-human immunocytochemistry (red) revealed that indeed, both EphA7-FL-HA and EphA7-T1-myc robustly bind to ephrin-A5. For C-E, nuclei were detected with Hoechst staining (blue) (Scale bars for all panels represent 30μm). (TIF) [file pone.0231561.s002.tif]

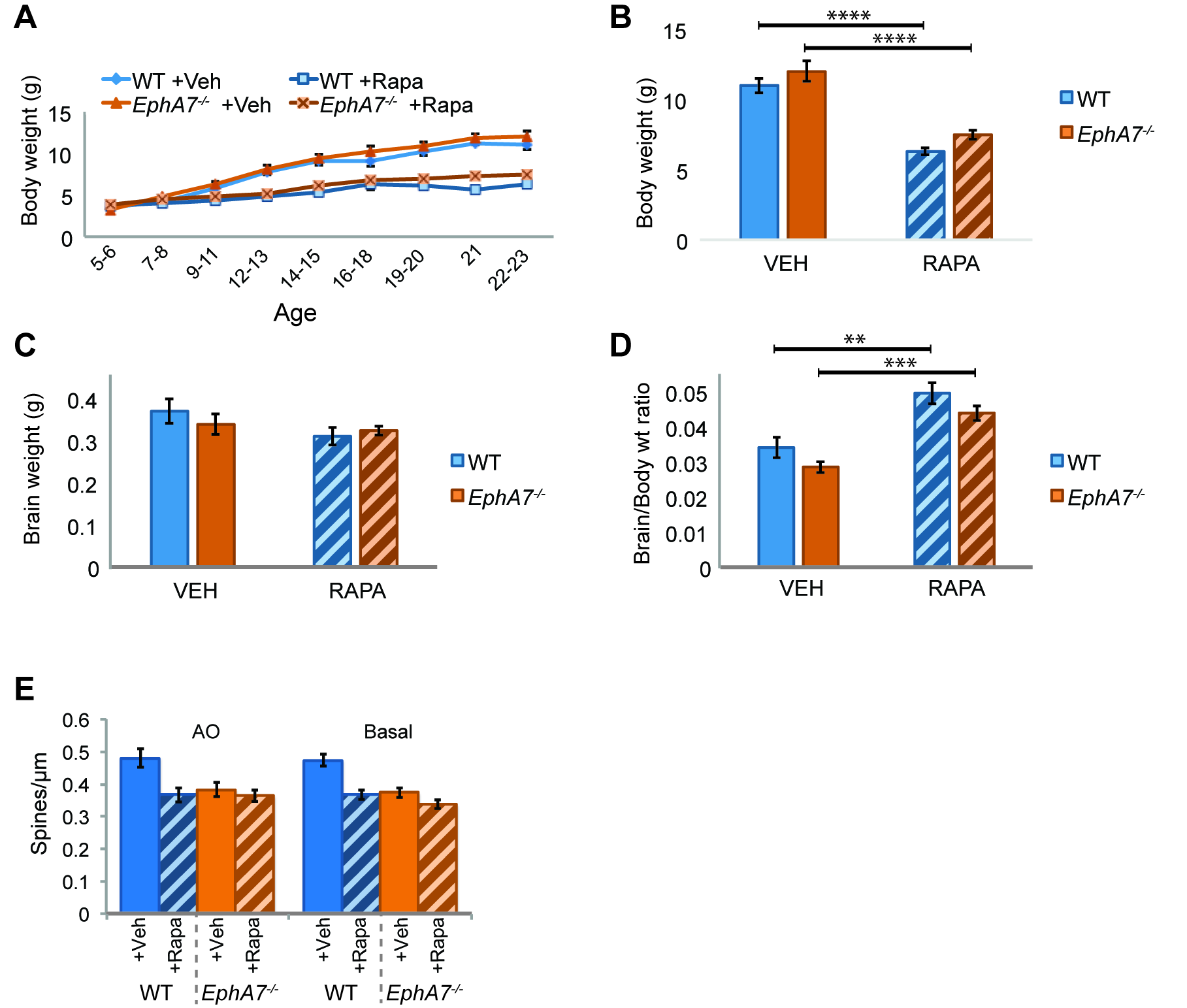

Supplement: S3 Fig — A. Growth curves of WT/vehicle (dark blue, n = 7), WT/rapamycin (light blue, n = 8), EphA7-/-/vehicle (dark orange, n = 9), and EphA7-/-/rapamycin (light orange, n = 10) animals. B. WT animals treated with rapamycin (6.29g ± 0.25g, blue stripe) weighed less than WT animals treated with vehicle (10.99g ± 0.53g, blue, p<0.0001) and EphA7-/- animals treated with rapamycin (7.49g ± 0.32g, orange stripe) weighted less than EphA7-/- animals treated with vehicle (11.98g ± 0.73g, orange, p<0.0001). There were no differences due to genotype. C. There were no differences in brain weight between WT/vehicle (0.37g ± 0.03g), WT/rapamycin (0.31g ± 0.02g), EphA7-/-/vehicle (0.34g ± 0.02g), and EphA7-/-/rapamycin (0.32g ± 0.01g) animals. D. The average brain weight to body weight ratio increased in rapamycin-treated WT (0.050 ± 0.003, blue stripe) and EphA7-/- (0.044 ± 0.002, orange stripe) animals compared to vehicle-treated WT (0.034 ± 0.003, blue) or EphA7-/- (0.029 ± 0.002, orange) animals. There were no differences due to genotype. E. There were no differences between dendritic spine densities counted from apical oblique (AO) versus basal dendritic branches, therefore total dendritic spine density is reported in Fig 3. (**p<0.01, ***p<0.001, ****p<0.0001). (TIF) [file pone.0231561.s003.tif]
