# Supplementary material for: EphA7 isoforms differentially regulate cortical dendrite development
Source: PLoS One. 2020 Dec 4;15(12):e0231561. doi: 10.1371/journal.pone.0231561 (PMC7717530; doi:10.1371/journal.pone.0231561)
Supplement: S1 Raw Images — (PDF) [file pone.0231561.s004.pdf]

Raw Image Files for Leonard, et al.

Figure 4A

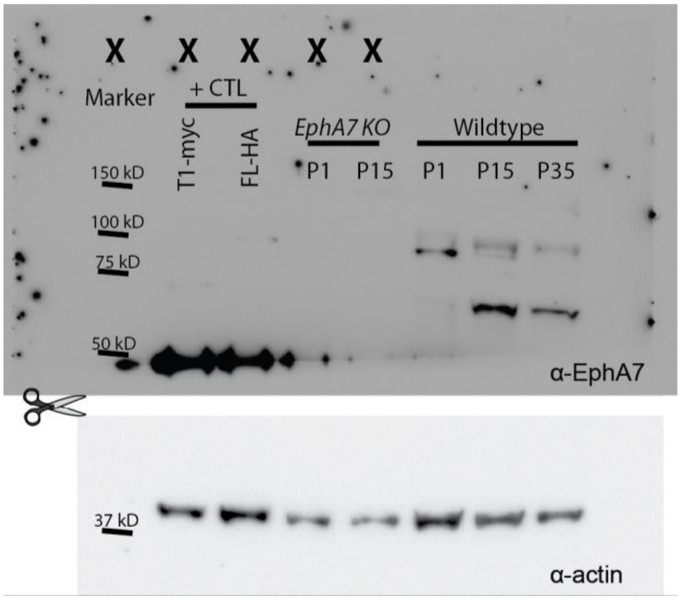

“+ CTL” = positive control from transfected cortical neuron lysate collected at DIV14.

“EphA7 KO” = negative control lysates from *EphA7*$^{-/-}$ cortex.

This membrane was cut at approx. 50kD to reduce the brightness of prominent background band that occurs w/ this antibody at this size. The bottom portion of the membrane was simultaneously probed for actin.

Digital image files acquired using chemidoc imaging system.

Figure 4B

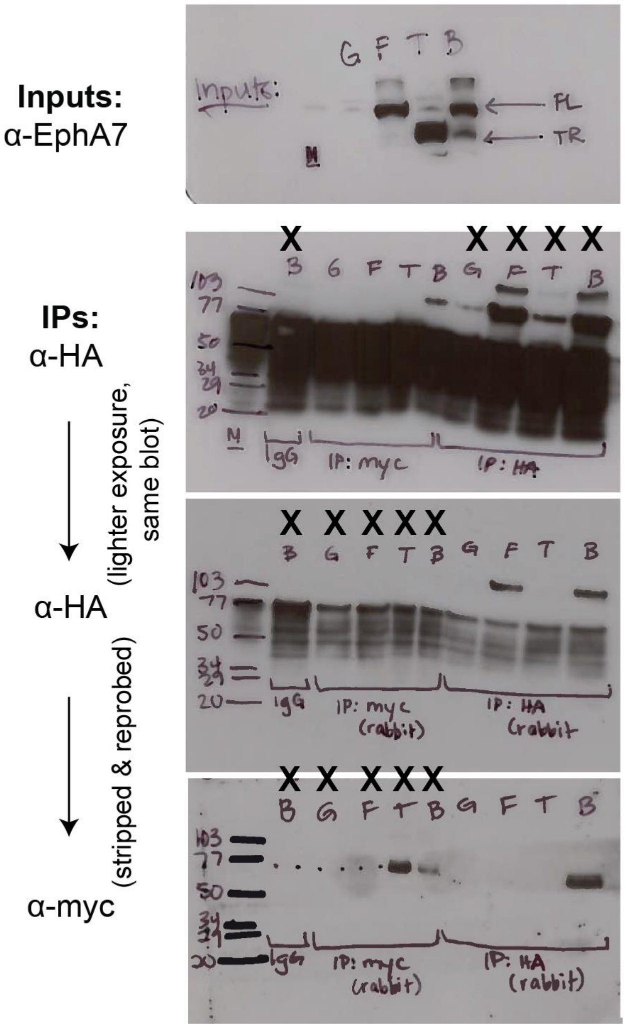

Sample Key:
G= GFP-only control
F= EphA7-FL transfection
T= EphA7-T1 transfection
B= both FL and T1 EphA7 transfection

“IgG” = negative IP control with rabbit IgG.

Digital scans from film exposures.

Figure 4C

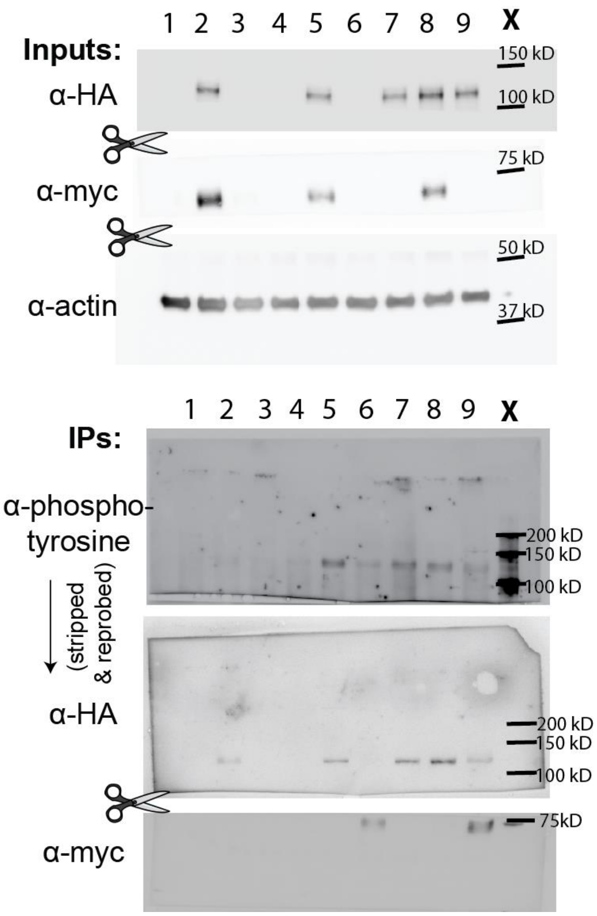

These blots were cut at the designated sizes, marked by scissors symbol, to simultaneously probe the same samples/membrane for multiple antigens.

Lanes:

1- Human-FC treatment (Control, ephrin-A5 stimulation). Control transfection (GFP only)
2- Human-FC treatment. EphA7-FL-HA transfection.
3- Human-FC treatment. EphA7-T1-myc transfection.
4- Ephrin-A5-FC treatment. Control transfection.
5- Ephrin-A5-FC treatment. EphA7-FL-HA transfection.
6- Ephrin-A5-FC treatment. EphA7-T1-myc transfection.
7- Ephrin-A5-FC treatment. FL-HA:T1-myc transfection, ratio 0.2:1.
8- Ephrin-A5-FC treatment. FL-HA:T1-myc transfection, ratio 1:1.
9- Ephrin-A5-FC treatment. FL-HA:T1-myc transfection, ratio 5:1.

Last - Marker

Digital image files acquired using chemidoc imaging system.

Figure 4D

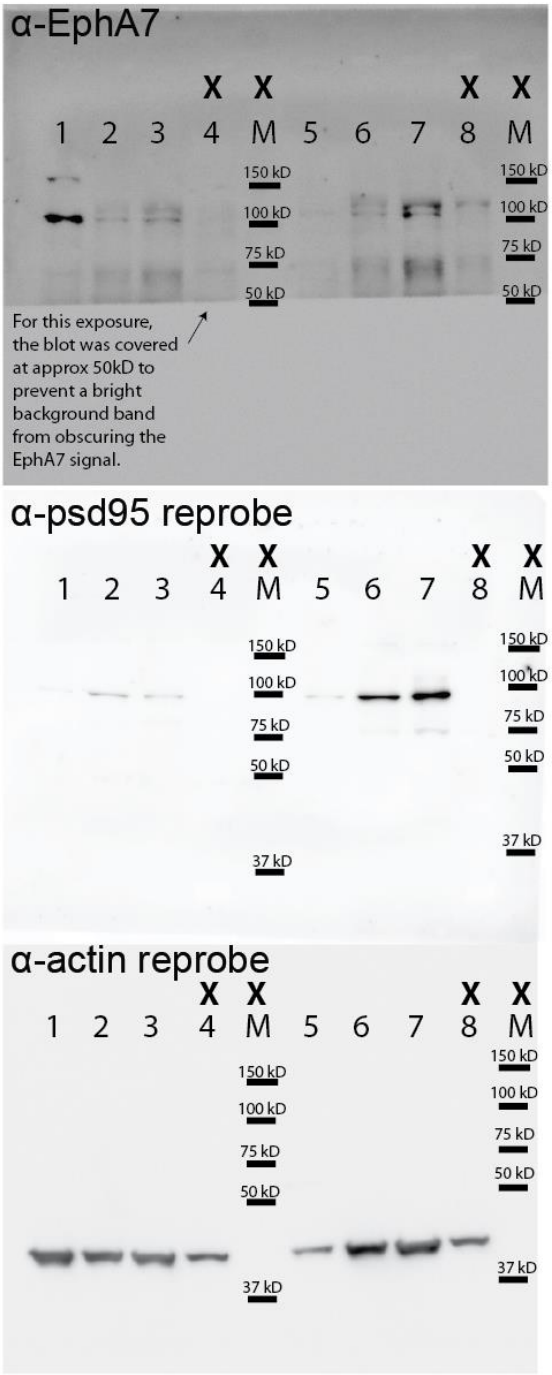

Lanes:

1- E18 rat cortex homogenate.
2- P2 rat cortex homogenate.
3- P10 rat cortex homogenate.
4- Lysate from DIV14 cortical neurons. (Signal was too low.)
5- E18 rat cortex synaptic fraction.
6- P2 rat cortex synaptic fraction.
7- P10 rat cortex synaptic fraction.
8- Synaptic fraction DIV14 neurons.
M- Marker

Digital image files acquired using chemidoc imaging system.

Figure S2

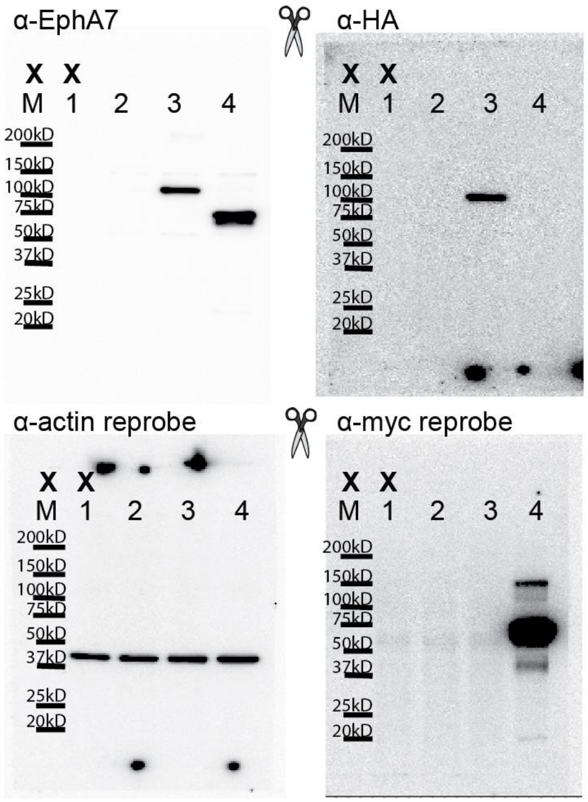

Lanes:

1- Untransfected HEK cell lysate.
2- Control transfection (GFP only).
3- EphA7-FL-HA transfection.
4- EphA7-T1-myc transfection.

Samples were run in duplicate and membranes were cut down the middle, designated by scissors symbol, to detect multiple antigens simultaneously.

Digital image files acquired using chemidoc imaging system.
